# Remembering Ralph Grassmann (1957 – 2008)

**DOI:** 10.1186/1742-4690-5-71

**Published:** 2008-08-04

**Authors:** Klemens Pichler, Kuan-Teh Jeang

**Affiliations:** 1 Institute of Clinical and Molecular Virology, University of Erlangen-Nuremberg, Erlangen, Germany; 2 Laboratory of Molecular Microbiology, NIAID, NIH Bethesda, Maryland 20892, USA

## Abstract

Friends and colleagues of Ralph Grassmann write their remembrances.

## 

July 1^st^, 2008, the retrovirology community lost an esteemed colleague and a friend. Ralph Grassmann (Fig. 1) passed away prematurely at age 50 after a courageous battle with kidney cancer. Ralph Grassmann studied biology at the University of Erlangen-Nuremberg. In 1985, he finished his diploma thesis, which investigated papillomavirus gene expression in various tumors of the skin, in the lab of Herbert Pfister. During his PhD in the group of Bernhard Fleckenstein, he focused on designing vector systems based on herpesviruses. Together with William Haseltine from Harvard University, Ralph tried and succeeded in using herpesviral vectors to express HTLV-1 proteins in T lymphocytes. These experiments demonstrated that HTLV-1 Tax was sufficient to immortalize T cells. In 1990, Ralph became a group leader and later received the Robert-Koch Förderpreis (Robert-Koch advancement award), which is awarded biennially to a young scientist excelling in virology, immunology or microbiology. He continued working on the molecular pathogenesis of HTLV-1 and became a professor in 2000 at the Institute of Clinical and Molecular Virology in Erlangen. Ralph was also a founding editorial board member of *Retrovirology*.

**Figure 1:**
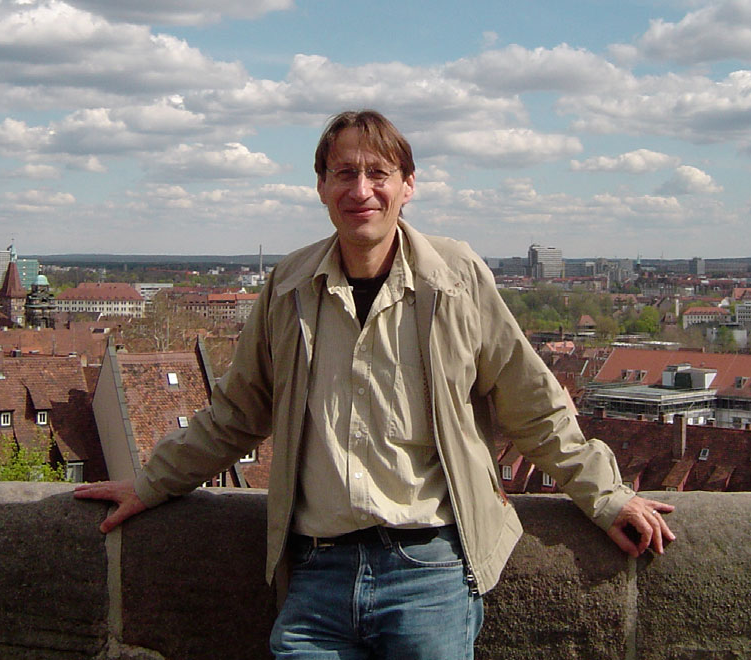
Ralph Grassmann, circa 2004.

We have collected here some remembrances of our friend Ralph, to honor and celebrate his life, his achievements, and to express our condolences to Ralph's wife Brigitte Grassmann-Sendelbeck and his sons, Thilo, Stephan and Mathias.

"Ralph was the one who introduced me to virology. Despite that I have worked only with soil bacteria before, he accepted me into a virology graduate training program. Of course, I needed lots of advice and he managed to squeeze me in whenever experimental details had to be discussed, even if his schedule was tight. Distilling the positive – the often hidden insights which is needed to continue the experiments – out of inconclusive results was one of his virtues that I will always remember. He was good at instilling the enthusiasm he felt about science in others – for instance, when we described the upregulation of a costimulatory receptor in HTLV-transformed lymphocytes by HTLV-1 Tax. Right until the end, Ralph's primary concern was the well-being of the people in his research group. The whole HTLV lab misses his counsel and supervision already". (Klemens Pichler)

"I met Ralph Grassmann the first time as an undergraduate student when he gave a lecture on tumor viruses. He was really motivating me with this interesting topic so that I decided to do my diploma and PhD thesis in his lab. Ralph was a fantastic group leader, who was always open to new ideas. Whenever I showed him my latest results, he always made the most of it. Although the results were not as expected at all times, he was very optimistic and gave me new input. After discussions and meetings with him, I was really motivated and enthusiastic to proceed. For his support, I cannot thank him enough. I can count myself lucky that Ralph was my supervisor. The passing of Ralph leaves scars in our group. We will miss his friendly character and his helpful suggestions". (Andrea Kress)

"In 1990, I started my PhD in the laboratory of Ralph Grassmann, working on HTLV-1 Rex and the Rex response element. We characterized the Rex binding site *in vitro *and *in vivo*. Additionally, we could show that the HTLV-1 Rex protein induces nuclear accumulation of unspliced viral RNA by avoiding intron excision and degradation. At the end of my PhD, I became pregnant. I still very much appreciate the way Ralph helped me to finish my doctoral thesis at that time". (Monika Gröne)

"I remember Ralph as a very nice colleague and friend, who was one of the first to warmly welcome me in the Institute, ten years ago, and the one giving me helpful support even until recently. During all this time, there was his constant attempt to cooperate and interact with each other without needing sharp elbows – a personal quality, which has become rare in the competitive environments of research. Ralph undoubtedly loved to work in the academic field, together with his research group and with all the students surrounding him, in order to perform a creative job and to develop his scientific concept. Surprisingly, nothing seemed to have become routine for him (as it could have been after many years of a hard struggle to establish himself and his research group permanently) but also little items were still worth for him to be discussed. We were happy to have Ralph as a very friendly partner and as a highly competent virologist. I will never forget the positive attitude he sent out and I hope to be able to carry forward some of his determinedness in science and in personal life". (Manfred Marschall)

"Ralph was an internationally respected scientist who combined research competence with human values. All who communicated with him have always been impressed by his open mind, his kindness and sincerity, and an aura of honesty. He was friendly, straightforward, conscientious, and with an intense sense of responsibility for his students. All of his friends and colleagues are in deep mourning. His death is a severe loss for the worldwide leukemia research community". (Bernhard Fleckenstein)

"I am surprised and deeply saddened by the news of Ralph Grassmann's death from kidney cancer earlier this month. I have followed Ralph's works for many years starting from the demonstration of HTLV-1 Tax as a transforming protein in T cells using a Herpesvirus saimiri vector to the more recent papers on Tax induction of anti-apoptotic protein HIAP, and Tax interaction with CDK4. Ralph's works had always been top-notch and of great impact. It is a great loss to our field that Ralph should die in the prime of his career. My thoughts go to his family and loved ones. On a personal note, I had the chance of talking to Ralph in depth during a meeting in Heidelberg in 2005. As that was my first trip to Germany, Ralph was ever so kind in helping me map out an itinerary from Heidelberg to Munich; and in giving me all sorts of pointers on where to go and what to see. He also kindly extended an invitation for me to visit him in Nürnberg. Unfortunately, I was not able to make the trip at the time. Now I really regret not having taken Ralph up on his kind offer. I believe this is very typical of Ralph who possessed such a quiet intensity and was always most kind and generous. Ralph will be greatly missed". (Joe Giam)

"I feel shocked at the news of Ralph's death. So far, I have not had any message from his university at Erlangen, Germany. Not long ago, in 2007 we all met at Hakone enjoying scientific discussions and friendship. I always feel that the HTLV meeting is like a big family meeting where all people share ideas, thoughts and sympathy. I was very pleased that Ralph was elected member of the executive committee in Jamaica. He did a good job promoting the European contribution at our international association. Maybe, nobody can substitute for his kind way to get involved with other people, but we shall remember him as a member of our big family". (Bernd Kitze)

"He was a very nice person to talk with and a good collaborator, he was ethical and very smart. I remember, once I provided to him some cell lines derived from ATLL labeled with the name of some patients I took care of. He thought that it was code name and presented at one HTLV1 meeting his work with the full name of these cell lines. I told him that it was not a code name and he was sincerely sorry and fixed this mistake very rapidly and afterwards we laughed at it. Best to his close friends and family". (Olivier Hermine)

"I am truly saddened to see one our rising stars to have his life ended so rapidly. Times like this I am especially affected since we work on basic biology of cancers and I feel helpless to see that our closest colleagues are affected and our research directly does not help or assist their survival. Perhaps events like this will push us for a speedier research in understanding various modes of cancers and defining better therapeutics or vaccines for a cure. I have known Ralph for more than 10 years and he was a good friend who also was interested in similar topics as my lab works on, including HTLV and cell cycle. We often discussed issues related to tax-deregulation of check points and anti-apoptotic machineries that are deregulated in HTLV infected cells. He was always a genuine and interested scientist in not only his own results but other peoples as well. I remember quite a few evenings of discussion over beer at Cold Spring Harbor as well as various HTLV-1 international meetings. I was especially touched by his cyclin work and his tax inducible experiments, where he not only would see the immediate results but also could accurately predict events downstream, which turned out to be true a few years later. That made him a visionary in our field and one that was not afraid of taking chances and risks to move our understandings beyond papers or grants. I will always remember him with fond memories. I also think we should start an award in his honor to be presented to the best "junior talk" at our regular HTLV international meetings and make sure that we don't forget our friend and colleague for many years to come". (Fatah Kashanchi)

"I was first introduced to Ralph through his ground-breaking papers in the late 80's on HTLV-1 Tax transformation using a rhadinovirus vector, but I didn't actually meet him until several years later at a Cold Spring Harbor Retrovirus meeting. I was immediately struck by his impeccable approach to science, his thoughtful consideration of ideas, and the wide beaming grin that would break through his serious expressions at any given moment. Over the years we met at numerous meetings and I considered him a friend, occasionally a competitor, but always an admired colleague. His creative work contributed enormously to the Tax field and I will miss him greatly". (Susan Jean Marriott)

"It was a shock to hear about Ralph's decease. I remember Ralph as a gentle and very friendly man, he was really pleasant company. He introduced me into the gene regulation of HTLV, a topic of interest to me since I was then studying gene regulation of HIV. He was an excellent scientist, and I visited him a few times in Erlangen. He will be dearly missed as a person and as a scientist". (Anne-Mieke Vandamme)

"Dear Ralph, Since we met more than 15 years ago, I remember a long list of very pleasant souvenirs from our relationship: jogging onto the Golden Gate in San Francisco, walking in the botanic garden in Rio de Janeiro, eating sushi in Tokyo, drinking a bit too much "smoked beer" in Bamberg, ... These memories will persist ... Memories will also remain through your contribution to the scientific community. You were one of the very few scientists having expertise in a broad range of areas: retroviruses, herpesviruses, plants and even birds. Your contribution to the mechanisms of cancer will definitely help to cure this terrible disease. But there is one characteristic of your personality that I recently discovered: your great dignity. I am very much impressed by your attitude while you were facing your illness. I noticed quite early that something was going wrong but, when I asked what was happening, you simply replied that you did not want your friends remembering that you were (very) sick and that you just decided to combat alone. I am not sure that I'll ever have your courage". (Luc Willems)

"Ralph Grassmann will be best remembered by the human retrovirology community for his contributions to the understanding of human T-cell leukemia virus type 1 (HTLV-1) pathogenesis. I first met Ralph at the Cold Spring Harbor RNA Tumor Virus meeting in 1989. At the time we were both doing postdoctoral fellowships in human retrovirology. In those early years we had very similar research interests trying to understand the mechanism of cellular transformation by HTLV. Ralph's unique studies with *Herpes saimiri *expressing the HTLV-1 *tax *gene provided some of the first evidence that Tax encodes the functions of HTLV-1 that immortalize primary human T-lymphocytes. At Cold Spring Harbor in 1991, I recall we even shared the stage to present and field questions on our independent findings on the functional and biochemical properties of HTLV Rex/RxRE interaction. Ralph was a great colleague and collaborator and throughout the years we exchanged many reagents and hypotheses regarding the regulation of HTLV-1 replication and pathogenesis. In 2005, Ralph invited me to visit Erlangen to present our most recent research and to interact with his University colleagues. Ralph was a fantastic host and tour guide and while visiting parts of Germany together it became clear that our respect for each other as colleagues over the years had developed into a memorable friendship". (Patrick Green)

"I remember Ralph first as a friend and second as a scientist. As a scientist Ralph never shrank from the difficult questions and in fact seemed to enjoy challenges that others generally avoided. Thus, in the pursuit of his science interest he has provided our community with significant research models and systems and answered several vexing HTLV questions. He accomplished these feats by independently struggling away or by just as easily forming a productive collaboration. Ralph envisioned collaborative "big" science well before it became the invention of necessity it is today. I and others have benefited from these collaborations and we will dearly miss him and these interactions. As a friend, Ralph was always ready with a warm greeting and a readiness to talk about any manner of things personal, political, or other. He was one of the first persons I met at my inaugural CSHL Retrovirology meeting as a post-doc and was someone I looked forward to reconnecting with every year thereafter. I will miss you Ralph". (John Semmes)

"When I heard of Ralph's passing on July 1^st^, the one immediate phrase that entered my head was that 'Only the Good Die Young'. Indeed, I then wrote to a colleague that knowing Ralph taught me that the most important thing in science is to be a good person. This was Ralph's seminal quality which we will all remember; and it transcends publishing 'big' papers, or being invited to give 'big' talks, or receiving 'big' awards that no one can recall even just a few years later. Ralph, Pat Green, Luc Willems, John Semmes, Fatah Kashanchi, Joe Giam, Susan Marriott and I, all came into the HTLV-1 field at about the same moment. The first time I met Ralph was at an early Cold Spring Harbor meeting with John Semmes. It must have been a very early meeting because at that time Ralph was absolutely convinced that I was John Semmes' postdoc and not the other way around. (I never did ask Ralph whether John Semmes had anything to do with convincing him that way). Nonetheless, it was one of those fateful encounters which led to many years of close scientific collaboration. Ralph and I published six papers together. I regard the work that he and I did on Tax immortalization of primary human T-cells [1] and Tax's effect on G1 cell cycle checkpoint [2] to be some of the more important findings in our field. Ralph also became one of my biggest supporters. When I first started *Retrovirology*, most of my friends (including Ralph) were skeptical; however, unlike others who simply sat on their hands, Ralph step forward early and contributed an excellent article to *Retrovirology *[3]. This was not his vote of confidence for the journal, but it was his vote for his friend, me. Indeed, toward the very end of his days when many other more important things should have occupied him, Ralph was diligently helping me finish the writing of a review article on microRNAs [4] which will be formally published after his passing. 'Hey, Ralph, if you are looking down from above, in this year that we hold a historical election in the United States, I vote for you; and I will always remember your friendship"'. (Teh Jeang)

## Authors' contributions

Both KP and KTJ wrote the article together. All authors read and approved the final manuscript.
